# Report of the 14^th^ Genomic Standards Consortium Meeting, Oxford, UK, September 17-21, 2012.

**DOI:** 10.4056/sigs.4319681

**Published:** 2014-03-20

**Authors:** Neil Davies, Dawn Field, Linda Amaral-Zettler, Katharine Barker, Mesude Bicak, Sarah Bourlat, Jonathan Coddington, John Deck, Alexei Drummond, Jack A. Gilbert, Frank Oliver Glöckner, Renzo Kottmann, Chris Meyer, Norman Morrison, Matthias Obst, Robert Robbins, Lynn Schriml, Peter Sterk, Steven Stones-Havas

**Affiliations:** 1 Gump South Pacific Research Station, University of California Berkeley, BP 244 98728 Moorea, French Polynesia; 2 Biodiversity Institute, Department of Zoology, University of Oxford, The Tinbergen Building, South Parks Road, Oxford, OX1 3PS, United Kingdom; 3 Centre for Ecology and Hydrology, Maclean Building, Benson Lane, Crowmarsh Gifford, Wallingford, Oxfordshire, OX10 8BB, United Kingdom; 4 Oxford e-Research Centre, University of Oxford, 7 Keble Road, Oxford, OX1 3QG, United Kingdom; 5 The Josephine Bay Paul Center for Comparative Molecular Biology and Evolution, Marine Biological Laboratory, Woods Hole, Massachusetts, USA; 6 The Office of the Associate Director for Science, National Museum of Natural History, Smithsonian Institution, MRC-106, 10th and Constitution Avenue N.W. Washington, DC 20560, USA; 7 Department of Biological and Environmental Sciences, University of Gothenburg, Box 463, SE-405 30 Gothenburg, Sweden; 8 Berkeley Natural History Museums, 1007 Valley Life Sciences, University of California, Berkeley, CA 94720, USA; 9 Department of Computer Science, University of Auckland, Auckland, New Zealand; 10 Allan Wilson Center for Molecular Ecology and Evolution, University of Auckland, Auckland, New Zealand; 11 Institute for Genomic and Systems Biology, Argonne National Laboratory, 9700 South Cass Avenue, Argonne, IL 60439, USA; 12 Department of Ecology and Evolution, University of Chicago, 5640 South Ellis Avenue, Chicago, IL 60637, USA; 13 Microbial Genomics Group, Max Planck Institute for Marine Microbiology, D-28359 Bremen & Jacobs University Bremen, Germany; 14 University of Manchester, Oxford Rd., Manchester, United Kingdom; 15 University of California San Diego, La Jolla, California USA; 16 Institute for Genome Sciences, University of Maryland School of Medicine, Baltimore, MD 20742, USA; 17 Biomatters Ltd., PO Box 5677, Wellesley Street, Auckland, New Zealand

## Abstract

This report summarizes the proceedings of the 14^th^ workshop of the Genomic Standards Consortium (GSC) held at the University of Oxford in September 2012. The primary goal of the workshop was to work towards the launch of the Genomic Observatories (GOs) Network under the GSC. For the first time, it brought together potential GOs sites, GSC members, and a range of interested partner organizations. It thus represented the first meeting of the GOs Network (GOs1). Key outcomes include the formation of a core group of “champions” ready to take the GOs Network forward, as well as the formation of working groups. The workshop also served as the first meeting of a wide range of participants in the Ocean Sampling Day (OSD) initiative, a first GOs action. Three projects with complementary interests – COST Action ES1103, MG4U and Micro B3 – organized joint sessions at the workshop. A two-day GSC Hackathon followed the main three days of meetings.

## Introduction

The Genomic Standards Consortium (GSC) formed in 2005 to tackle the challenge of working as a community towards improving the quantity and quality of contextual data made accessible for genomes and metagenomes [1]. The GSC works towards its goal through the creation, maintenance, and adoption of a range of standards and collaborative projects. Further information about the GSC and its range of activities can be found on the GSC wiki pages [2]. Here we describe the proceedings and outcomes of the GSC’s 14th meeting (GSC14) at Oxford University, 17-19 September 2012.

The main theme of GSC 14 was “genomics enabled long-term, place-based research”. A core mission of the GSC is to improve the contextualization of genomic data, and perhaps the best chance to undertake highly contextualized genomic research over the long-term is to leverage places that are already the focus of sustained ecological, environmental, and social research. Discussion of a GSC focus on “place-based” genomic research began at the GSC11 meeting in Cambridge, UK in April 2011 and continued at the GSC 12 meeting in Bremen, Germany in September that same year. These discussions led to a call for an international network of “Genomic Observatories” from scientists associated with various intensively studied sites around the world [3]. The GOs concept was further developed at the GSC’s 13th meeting in Shenzhen, China in March 2012 [4]. GSC 14 represented the first formal meeting of the emerging GOs Network, bringing together the GSC community, potential GOs sites, and GOs partner organizations to identify shared interests and consider strategies for the future.

The meeting was structured into six sessions that aimed to define the GOs Network mission, outline some initial actions, and explore options for its organizational structure. Workshop objectives included to (i) present candidate GOs sites and GOs partner organizations, (ii) discuss GOs actions, (iii) develop interactions between GOs and the broader GSC community, and (iv) give the participants time to form working groups and deliberate priorities. The first session that introduced the work of the GSC was co-organized and supported by the European Cooperation in Science and Technology (COST) Action (ES1103) [5]. Following the main meeting, two satellite meetings were organized and supported by EU FP7 projects. The first, Marine Genomics for Users (MG4U) [6] was a stakeholders session considering how to translate advances in genomic research to ecosystem-based marine management actions and policy perspectives. The second satellite was a planning meeting of the Marine Biodiversity, Bioinformatics, Biotechnology (Micro B3) project [7] that leads Ocean Sampling Day, one of the first GOs actions. A two-day GSC hackathon also followed the meeting, which focused on issues related to the GOs informatics stack. The workshop presentations as well as interviews with some of the attendees were recorded by Mediomix (http://mediomix.de/) and are available on YouTube (http://www.youtube.com/playlist?list=PLgacjRIHqvMD6H1eJaD0iHzyoANpc2L4l).

The 15th Genomic Standards Consortium (GSC 15) meeting will be held at the National Institute of Health (NIH) in Bethesda, Maryland, 22-24 April 2013. This meeting will highlight the utilization of genome metadata standards for the enhancement of our biological understanding of microbes, the interaction between microbial genomes, human health and disease. It will include a GOs Network session (considering the potential of citizen science genomics and humans as GOs) and a GOs satellite meeting at the Smithsonian Institution (focused on marine genomic observing systems and Ocean Sampling Day). Please see the GSC 15 home page [8] for further details.

## Session I: GSC Standards - Introduction and updates

Chaired by Peter Sterk (University of Oxford, UK), the opening session served to familiarize newcomers with the GSC and the range of its activities. It was hosted as a joint session with the Microbial Ecology & the Earth System [5] consortium, a European Cooperation in Science and Technology (COST) action. The session introduced new participants to the GSC and explored how to work with the GSC standards.

Dawn Field (NERC Centre for Ecology and Hydrology Wallingford, UK; University of Oxford, UK) introduced “An Open Call to work under the umbrella of the GSC” describing the background of the GSC and announcing its new policy for initiating projects. The GSC is an open membership community working towards better-standardized descriptions of genomes, metagenomes and marker gene sets [1]. Standards are key to accelerating science, avoiding redundancy and enabling integration of data streams. The GSC runs a range of consensus-driven projects and is calling for community involvement and encouraging broader compliance. The GSC welcomes the involvement of the GOs Network as a GSC Project/Working Group and looks forward to future interactions. Among other things, the GOs Network will provide *use-cases* for further developing GSC standards and *showcases* for the scientific opportunities that flow from their application.

Renzo Kottmann (Max Planck Institute Bremen, Germany) then described the background and mission of the “Microbial Ecology & the Earth System” COST action. He introduced the microbial ecology workflow from field sampling to analysis and publication and the need for standards in this domain. He explained how the GSC’s Minimum Information about any Sequence (MIxS) standard specifically covers the “xyzt” metadata (latitude, longitude, depth and time) that are fundamental to field biology. Pelin Yilmaz (Max Planck Institute Bremen, Germany), the lead author on the GSC’s MIxS publication, then gave a detailed description of the development of the MIxS standard and its use today [9].

Seven short talks covered aspects of the emerging “Microbial Ecology Data Pipelines”. The first presentation was by Steven Stones-Havas (Biomatters Ltd., New Zealand) describing work to connect Field Information Management Systems (FIMS) to Laboratory Information Management Systems (LIMS) and specifically the FIMS/LIMS developed for the Moorea Biocode Project [10]. One product of this work is the open-source Biocode LIMS - a free plugin for DNA barcoding on the Geneious software platform [11]. The next talks focused on analysis of data and included an update on the newly launched metagenomics portal (MG Portal) [12] at the European Bioinformatics Institute (Sarah Hunter, European Bioinformatics Institute, UK), an update on CAMERA [13] (Jeff Grethe, University of California San Diego, USA), and presentation of the BIOM standard as a new addition to QIIME [14] (Jai Ram Rideout, University of Colorado, Boulder, USA). Anna Klindworth (Max Planck Institute Bremen, Germany) then talked on the evaluation of primer and primer pairs for 16S ribosomal RNA biodiversity studies. Finally, Guy Cochrane (European Bioinformatics Institute, UK) outlined submission of MIxS-compliant data to the International Nucleotide Sequence Database Collaboration (INSDC) [15] and provided some insight into the challenges for those designing and operating submission services. George Garrity (Michigan State University, USA) finished the session with an update on the GSC’s open access journal “Standards in Genomic Sciences” (SIGS) [16]. Overall, the presentations highlighted the importance of consistent generation and flow of MIxS compliant data through Microbial Ecology Data Pipelines from field sampling to analysis and publication. The MIxS standard adds value to the sequence data and better ensures comparability of different sequence data sets.

## GOs Sites and GO Network Partners (Sessions II & IV: Lightning talks)

GSC 14 introduced two sets of key participants interested in joining or collaborating with the GOs Network: (a) representatives of intensively studied sites (Session II, Table 1) and (b) organizations, networks, and initiatives interested in partnering with GOs (Session IV, Table 2). The absence of some sites already engaged in the discussions of the GOs Network was noted, and it was also acknowledged that other very worthy sites had not yet been approached. All agreed that a broader geographic representation of GOs would be desirable in the future. Likewise, although a wide range of potential partners was represented, some were absent and others that should be involved in the founding of the network have not yet been contacted.

**Table 1:** Candidate Sites with their GSC 14 representatives/authors

**Candidate Site**	**Representative**
*Asia-Pacific*	
Moorea, French Polynesia	Serge Planes (CNRS-EPHE CRIOBE) and Neil Davies* (UC Berkeley)
Hauturu, New Zealand	Alexei Drummond (Auckland)
*Europe*	
Rothamsted, UK	Penny Hirsch (Rothamsted)
Plymouth L4, UK	Tim Smyth (Plymouth)
Roscoff, France	Nathalie Simon (UPMC-CNRS SBR)
Helgoland, Germany	Gunnar Gerdts (Alfred Wegener Institut)
Naples MareChiara LTER, Italy	Adriana Zingone (SZN)
Crete, Greece	Georgios Kotoulas (HCMR)
Banyuls, France	Ian Salters (Banyuls)
	
*Polar*	
Ny-Ålesund Svalbard Arctic & Rothera Antarctica	Melody Clark (BAS)
	
*Americas*	
Northern Temperate Lakes LTER, USA	Trina McMahon (University of Wisconsin)
Western Atlantic, Smithsonian Marine Science Network	Chris Meyer (Smithsonian)
Southern California Coastal Waters, USA *Presenter	Steve Weisberg (SCCWRP)

**Table 2:** Candidate partners and their representatives at GSC 14

**Candidate partners**	**Representative**
*Networks of Sites*	
European Network of Marine Research Institutes and Stations (MARS) [17]	Michael Thorndyke (SLC Sweden); presentation given by Matthias Obst
U.S. Western Association of Marine Laboratories (WAML) [18]	Steve Weisberg (Southern California Water Research Program)
Smithsonian Global Earth Observatory (SI GEO) [19]	Eldridge Bermingham (Smithsonian)
Global Lake Ecological Observatory Network (GLEON) [20]	Trina McMahon (University of Wisconsin)
	
*Infrastructures*	
European Marine Biological Resource Center (EMBRC) [21]	Colin Brownlee (MBA)
Global Genome Initiative (GGI) [22]	Jonathan Coddington (Smithsonian)
Barcode of Life (IBOL, BOLD) [23]	Sujeevan Ratnasingham (Guelph)
Group on Earth Observations Biodiversity Observing Network (GEO BON) [24]	Dan Faith (Australian Museum)
	
*Institutes, Initiatives and Programs*	
Berkeley Initiative Global Change Biology (BIGCB) [25]	Michelle Koo (Berkeley)
International Soil Metagenome Sequencing Consortium & RCN (Terragenome) [26]	Folker Meyer (Argonne)
Biodiversity Virtual e-Laboratory (BioVeL) [27]	Matthias Obst (Gothenburg)
Earth Microbiome Project (EMP) [28]	Jack A. Gilbert (Argonne/Chicago)

Dawn Field (NERC Centre for Ecology and Hydrology Wallingford, UK; University of Oxford, UK) chaired the session and gave a brief overview of the GOs Network since the initiation of the concept following GSC 11 (April 2011) when she and Neil Davies began discussing the benefits of place-based genomic research at the “L4” and “Moorea” sites [4]. Subsequent discussions with an ever growing number of researchers pioneering DNA-based approaches at premier research sites, led to a letter published in early 2012 by 68 authors (many present at GSC 14) calling for the creation of a Genomic Observatories Network [3]. GSC 14 offered the first chance for this group to meet and decide the future of the GOs Network.

The candidate sites (potential Genomic Observatories) all represented long-term research sites with decades to a century of data thus offering unique opportunities to integrate genomic research into a range of other research activities. The range of candidate partners included networks of sites, institutions (field stations, marine labs, universities, research institutes), and organizations/programs that contribute to and benefit from place-based research in genomics (*e.g.,* by providing essential contextual data and/or research infrastructure). Each site and partner was invited to give an overview of its history, research and future plans to work as a genomic observatory. Since talks were only 5 minutes, they were all structured into a common template with sections for (i) Genomic Observatories (Site Description, Institutional Partners, Facilities, Access to Biodiversity, Data Sets) and (ii) Genomic Observatories Partners (History and Structure, Mission, Assets, How (meta)data standards are employed, What you would gain from and contribute to a GOs Network). Subsequent to GSC14 all sites and partners will be invited to expand their presentation into short papers.

## Session III: GOs First Actions

The last session of Day 1 was the first opportunity at this meeting to have an open discussion about how best to build the Network. To drive discussions of what a GOs Network could contribute to science, the Ocean Sampling Day (OSD) initiative [29], which involved several candidate GOs sites, was discussed as a case study. Two brief presentations were given to set the stage for the discussions. First, the OSD initiative was described by Frank Oliver Glöckner (Jacobs University Bremen & Max Planck Institute Bremen, Germany) in the context of the EU 7FP Micro B3 (Biodiversity, Bioinformatics, Biotechnology) project [7], which is leading OSD. Micro B3 will develop innovative bioinformatic approaches and a legal framework to make large-scale data on marine viral, bacterial, archaeal and protist genomes and metagenomes accessible for marine ecosystems biology and to define new targets for biotechnological applications. The project builds upon a highly interdisciplinary consortium of 32 academic and industrial partners comprising world-leading experts in bioinformatics, computer science, biology, ecology, oceanography, bioprospecting and biotechnology, as well as legal aspects. Ocean Sampling Day is a global, simultaneous megasequencing campaign that will take place on the June solstice 2014. OSD is designed to collect standardized data suitable for modeling the distributions, abundances and functions of microbes in the world’s oceans. A first pilot run, which leveraged the emerging GOs Network, took place on the solstice in June 2012.

The second talk presented crowd funding as a new way to support aspects of such international projects. Wolfgang Hankeln (Mediomix, Germany) made a short pitch for the OSD community to let his company create a movie about OSD funded by public contributions to the project.

The chair of the session, Chris Meyer (Smithsonian, USA) then invited all of the OSD participants at the meeting to join an open panel discussion. Participants included Georgios Kotoulas (Crete), Burak Ali Cicek (Cyprus), Gunnar Gerdts (Helgoland), Neil Davies (Moorea), Adriana Zingone (Naples), Nathalie Simon (Roscoff), Melody Clark (Rothera), Viggó Þór Marteinsson (Iceland), Tim Smyth (Plymouth L4).

Each participant in the pilot 2012 OSD was asked to self-sort their participation along an axis of ease in participation from 1 being difficult to 10 being very easy. This created a gradient of partners who found the sampling and processing easy to do with minimal extra effort, at one end, versus more challenging and significant additional effort, at the other. Most of the pilot sites characterized themselves well toward the “easy side” of the spectrum, with a few partners sprinkled down the chain. Discussions then focused on the difficulties encountered in order to better coordinate and prepare for a more extensive effort. OSD is a first example of the way that coordinated research projects (*e.g.*, Micro B3) might leverage the GOs Network. For example, many of the sites participating in the OSD pilot were not part of the Micro B3 grant. They contributed time and resources to make the collections because of their interest in developing common standards-based genomic datasets and in sharing their experiences with others in a mutual knowledge exchange (hence their participation in the GOs Network.

It was agreed that the common platform (instrument) the GOs Network could represent, especially when specifically proven and tested by initiatives such as OSD, would enable many other future projects involving coordination and comparable approaches across regions, and eventually the globe.

## Sessions V-VII: Breakout Groups and the formation of GOs Working Groups

A key aim of this meeting was to establish persistent mechanisms for building the network, to take forward actions, and establish shared best practices. This included discussing preferred governance models and establishing working groups to help build the GOs strategy. The breakout sessions were dedicated to the exploration of four topics and the governance of the GOs Network.

To set the scene for the breakout sessions, Robert Robbins (University of California San Diego, USA) chaired a session with presentations by the leads of each breakout group. Talks covered (i) Biodiversity Genomics, (ii) Theory and Modeling, (iii) Informatics (Biocode Commons), and (iv) Bioarchiving and Access and Benefit Sharing (ABS). For each topic, the chair gave a 20-minute “charge” talk and then the groups broke out to discuss, supported by a panel of experts in the topic. Day Three was dedicated to giving time for all the breakout groups to report back to the full group.

The Biodiversity Genomics group, chaired by Chris Meyer (Smithsonian, USA), was deemed so central to the GOs Network that the core group of the GOs Network was tasked to develop it further. Topics covered in discussion included in particular the biology of Dark Taxa and how this presented challenges for data acquisition and analysis. Are new approaches needed in taxonomy, especially as more microbial data are generated where unknown taxa (and genes) are routinely found? In subsequent cross-discussions between the working groups it became clear that developing the science of biodiversity genomics was a general goal of the GOs Network that would require input from all, but especially the Theory and Informatics working groups.

Following the meeting it was agreed that the GOs Network would take forward its activities under 5 key umbrella areas: **Science, Informatics, Technologies, Policy, and Governance.** The Theory & Modeling group would fall under Science, Biocode Commons under Informatics, ABS under the broader topic of Policy (including data management policies and the social/cultural challenges of applying biodiversity genomics to achieve policy goals, such as ‘good environmental status’). Governance would be formalized into the leadership of the GOs Network. The Technologies thematic area was added to cover Bioarchiving (handling and preserving samples from field collection to *ex situ* repositories) as well as the rapid evolution of molecular analytical approaches, such as *in situ* ecogenomic sensor platforms. This structure provides ample coverage of topics and will allow the GOs Network to grow areas as required. Coordination across these main thematic areas between groups will be the responsibility of the core group of the GOs Network - an informal steering committee - until and unless a more formal mechanism is agreed.

### SCIENCE: Theory & Modeling

Alexei Drummond (Auckland, NZ) chaired the session with panel members Alfried Vogler (NHM/Imperial), Rampal Etienne (University of Groningen), Dan Faith (Australian Museum), Jack Gilbert (Argonne National Laboratory), and minutes taken by Pier Luigi Buttigieg (MPI Bremen).

**Introduction**: Before the meeting, a list of key scientific questions was identified for discussion by the breakout group [1]: How to assess (observe) biodiversity patterns at different temporal and spatial scales [2]? What are the rules governing macro-ecological patterns [3]? What do these rules tell us about what will happen next given major human-induced perturbations of the Earth’s climate and ecosystems [4]? How do genomic technologies and place-based studies help?

Group chair Alexei Drummond (Auckland, NZ) pointed out in his opening presentation that ecology and genomic technologies have been on a” collision course” for a number of years and are really starting to hit home now. To date, molecular ecology has typically focused on a small number of species and their genetic variation (usually from a small number of loci) in space and time. Genomic Observatories, however, now offer the opportunity of studying much larger numbers of well-contextualized (e.g., with environmental sensors) genes (up to whole genomes), individuals, and species within and between ecosystems. Such capabilities provide challenges (e.g., how to treat such large heterogeneous data streams) as well as opportunities to advance ecological theory.

**Goals:** Identify the grand challenges in theory and modeling relevant to GOs

**Findings and Recommendations:** The group advised the GOs Network to base its future work on the fundamentals of good (scientific) theory, that is: (a) Consistent with observed phenomena, (b) Has predictive power, (c) Applicable to a broad range of phenomena, (d) Simple / parsimonious.

The kinds of macro-ecological observations and phenomena (data to be understood/ modeled) by the GOs Network was found to include:

- Species abundance distributions in an area (SADs)
- Species-area relationship (SAR) and Phylogenetic Diversity-area relationship
- Abundance-occupancy correlations (decay of diversity similarity with distance)
- Next-generation environmental sequence data and associated environmental measurements (temperature, humidity, soil chemistry)

The GOs Network, with its highly characterized sites, could make important contributions to major questions such as [1]: understanding the nature and significance of the long tail of rare taxa in ecosystems (at the species and the genetic level), and [2] assessing the importance of interactions within and among levels of biological organization (species, individual organisms, genes).

How to bring together macro-ecological theories that assume no significant interactions among species (e.g. [30,31].) with those that are explicitly based on interactions (e.g [32].) could provide an overarching challenge for the GOs Network to address. In any case, ecological network theory is likely to be very important for the GOs Network, which should represent a powerful source of data on:

- Consumer-resource body size ratios
- Metabolite/Resource use networks
- Distributions of connectivity
- Interaction strengths

Intensive study at GOs sites and use of genomic approaches is also likely to provide a wealth of valuable trait information, such as:

- Trait distributions of species in an area (e.g. biomass)
- Trait distributions of individuals in a species or community (e.g. body size)
- Distributions of community-level functional traits (e.g., through metagenomics)
- Species range size distributions

Given the likely evolution of the GOs Network and the types of data it will generate, the following questions should be developed at future GOs Network meetings:

1. Does greater diversity imply greater functionality and/or resilience? Can GOs Network address this (semi) quantitatively?
2. What kind of functions are we talking about? Are they readily measurable? How many species are present/are missing? The GOs Network could provide (more) solid data points from which one could extrapolate (if a coherent sampling design is in place).
3. What are the marginal losses/impacts of losing a given instance (gene, species, function) of an ecosystem - what is the marginal value of incremental increase/loss of biodiversity?

### INFORMATICS: Biocode Commons - the Informatics Stack for Genomic Observatories

John Deck (University of California Berkeley, USA) and Steven Stones-Havas (Biomatters Ltd., New Zealand) chaired the session, with panel members Renzo Kottmann (Max Planck Institute Bremen, Germany), Norman Morrison (University of Manchester, UK), Philippe Rocca-Serra (University of Oxford, UK), Mesude Bicak (NERC Centre for Ecology and Hydrology Wallingford, UK).

**Introduction:** The Biocode Commons aims to coordinate the interoperable and standards based “informatics stack” for the global network of Genomic Observatories. The GOs Network user requirements will provide the overarching strategic direction for the Biocode Commons and will serve as its major user community (although the scope of the Biocode Commons could expand in the future). The space between standards and software, standards and other standards, and between software and other software, is what we are calling the “commons” here and “Biocode Commons” specifically addresses the gap present in the biodiversity genomics community between collections-based systems (commonly museum specimens and tissues) and sequence-based systems (DNA and increasingly a range of other ‘omics data types).

**Goals, Findings, and Recommendations:** The goals for this session were to define the data types, data formats, software, standards, and governance structure for the Biocode Commons. The categories of data the group outlined as being relevant for the GOs Network included: meta, phenotypic, environmental, taxonomic, genomic, geographic, and sample. The group defined a list of many different formats for sharing data, including BIOM, ISA-tab, GCDML, ABCDDNA, INSDC structured formats, Darwin Core archives, to name but a few. Software was discussed and the group outlined several principles that software should adhere to including versioning, and creating both human and machine interfaces. Finally, the group suggested compiling a registry of Genomic Observatories (sites) and to enable local repositories to construct globally unique identifiers for all samples. Discussions are ongoing within the Biocode Commons [20].

## POLICY: Bioarchiving, Access and Benefit Sharing

The first foray into the general area of “policy” for the GOs Network dealt with the topic of access and benefit sharing (ABS). The session was chaired by Jonathan Coddington (Smithsonian Institution, USA) with participants Michèle Barbier (Mediterranean Science Commission, France), Katie Barker (Smithsonian Institution, USA), John Benzie (University College Cork, Ireland), Arianna Broggiato (Université Catholique de Louvain, Belgium), Neil Davies (University of California Berkeley, French Polynesia), Chris Hunter (European Bioinformatics Institute, UK), Anna Klindworth (Max Planck Institute Bremen, Germany), Chris Lyal (Natural History Museum, London, UK), Frank Oliver Glöckner (Max Planck Institute Bremen, Germany), Johanna Wesnigk (EMPA Bremen, Germany), Antje Wichels (AWI, Germany)

**Introduction:** Place-based genomic research requires sampling and sharing of physical biodiversity (samples/specimens), often among institutions and across national borders. These exchanges of biomaterials (and associated digital data and metadata) take place over periods of many years. This creates challenges that are both technical/biochemical (how to handle samples in the field in the short term, and how to transport and store them over the long term?) and legal (what are the terms of use for the biomaterials sampled - notably ABS - and the cascade of subsamples, data, and other products that flow from them?)

**Goals:** This session covered two topics: (i) Capturing Genomes (Sample handling and preservation protocols along the supply chain - from field to repository - for future ‘omics studies), and (ii) Access and Benefit Sharing (ABS) across GOs sites. A third relevant topic, Informatics (tracking materials and data from collection through analyses and publication) was already the theme of another breakout group (Biocode Commons).

**Findings:** Participants agreed that the main two topics are distinct and complex and should be the subject of separate working groups in the future. The participants thought that a third topic of “Ecogenomic Technologies” might form another future working group addressing how much can be automated *in situ* and how such mechanization (including robotics and ecogenomic sensors) would help standardization of protocols and tracking of metadata.

**Recommendations:** GOs should form separate working groups:

1 Access and Benefit Sharing Working Groups (ABS WG)
2 Capturing Genomes Working Group (CG WG), to include initially ‘Ecogenomic Technologies’

**Actions:** Coordinate with the Global Genome Initiative (GGI, see http://www.mnh.si.edu/ggi/) to host a workshop on Capturing Genomes at Smithsonian in conjunction with the upcoming GSC 15 meeting in April 2013.

The group then focused its main discussions on ABS and became effectively the first meeting of the GOs ABS Working Group.

**Deliverables:** The session participants recognized that the detailed text in each ABS agreement depends on the provider country that has ultimate jurisdiction (under CBD). Consequently, the text of ABS agreements is likely to vary somewhat from country to country, even if all essential aspects of a project are otherwise identical across GOs sites. Despite this inevitable complexity, a general ABS philosophy for the GOs network is important in helping establish trust and in guiding the text of specific agreements. Furthermore, template agreements for multi-site projects across the GOs network can help to facilitate the ABS process by providing useful text that will apply at all participating GOs. The ABS WG therefore agreed to collaborate on two deliverables:

1. *Code of Conduct for Genetic Resource Access and Use in the GOs Network*. This non-binding Memorandum of Understanding (MoU) would harmonize ABS philosophy across museums (GOs partners) and field stations, (GOs sites) the latter being key trusted partners in provider countries, the former being the primary institutions tasked with maintaining *ex situ* collections for the global scientific community. Input for this code of conduct will come from various sources, such as the Mediterranean Code of Conduct being initiated by CIESM (The Mediterranean Science Commission)
2. *Ocean Sampling Day (OSD) Use Cases -* As OSD is the first GOs action, the group will help draft the following OSD documents, providing specific examples for future projects:
  a A template *OSD ABS Agreement.* Guided by the Code of Conduct, the group will help draft a specific ‘template’ for OSD, initially aimed at non-commercial use. A subsequent version for commercial use will be prepared before December 31, 2012.
  b A template *OSD Material Transfer Agreement* for bioarchiving. The group will draft an example agreement for archiving OSD samples with OSD repositories. The example agreement will function as an agreement between each participating OSD partner and the Smithsonian Institution (NMNH), an OSD repository. A final version will be prepared in conjunction with the OSD ABS agreement.

The first versions of the documents have been created as part of the efforts of Micro B3’s workpackage 8 [33].

### GOVERNANCE: Building a long-term vision for the Genomic Observatories Network

**Introduction**: This session provided the chance for interested GSC 14 participants to consider governance models for the GOs Network. Going into the meeting, the GOs Network was incubated under the GSC as a GSC Project and it was agreed by the group that this working relationship is appropriate and should remain. Frank Oliver Glöckner (Max Planck Institute Bremen, Germany) chaired the session, which began with a brief introduction by Dawn Field (CEH Oxford, UK) on how the GSC is currently governed. The GSC has a Board of 20 academics who have taken on a leadership role in GSC projects. At the time of writing, fifteen projects now work under the umbrella of the GSC, including the GOs Network, and the Biocode Commons, and new project proposals are welcomed. The lead or leads of each GSC project are expected to complete a project description that should be updated annually and to advance activities through balanced working groups. It is expected that larger projects, like the GOs Network might eventually have more formal governance mechanisms (steering committees, etc). All projects working under the GSC umbrella are expected to contribute to the overall GSC mission, help to define GSC strategy, attend and help organize GSC events, and report on progress in a regular fashion to the GSC Board and to the broader GSC community. Project descriptions have already been drafted for the GOs Network, Biocode Commons and the “Minimum Information about a Genomic Observatory” (MIGO) specification and are available for comment.

For the rest of the session, participants then split into two groups to discuss the governance of the GOs Network as a whole and the Biocode Commons in particular (as the informatics arm of the GOs Network - see description above). Dawn Field (NERC Centre for Ecology and Hydrology Wallingford, UK; University of Oxford, UK) chaired the discussion of the GOs Network governance with panel members Neil Davies (University of California Berkeley, French Polynesia), Jack A. Gilbert (Argonne National Laboratory, USA; University of Chicago, USA), Matthias Obst (University of Gothenburg, Sweden), Chris Meyer (Smithsonian Institution, USA), Linda Amaral-Zettler (Marine Biological Laboratory, USA).

The GOs Governance breakout group report follows:

**Goals**: The first agenda item of the breakout session was to re-affirm, in the light of the previous sessions, that there was consensus among those present that the GOs Network should go forward. This was unanimously agreed upon. The second goal was to establish a mechanism of governance, no matter how informal, to take forward the Network post-GSC 14. To date, the GOs Network was promoted largely by the six Panel members of the session, who recognized that it should eventually have strong leadership drawn from many if not all represented GOs sites and partners. Key representatives from many related initiatives, especially large networks that may contain multiple GOs sites, should also be involved in some capacity in the future. The third goal was to work towards a mission statement and the creation of a Founding Charter.

**Recommendations**: It was agreed that the GOs Network exists ‘*de facto*’ as a result of the group-authored Nature Correspondence [3] and currently consists of a group of *individuals* with shared interests. Formalizing what is meant by “Genomic Observatory” and defining how/if individuals, sites, and partners should formally join the network would need to be developed in time. Membership in the Network was discussed at length and it was agreed that this was for the core group to work on over the coming months in consultation with the wider community. In particular, the group liked the GLEON [34] model of “grass-roots governance”, introduced by Trina McMahon (University of Wisconsin) with membership open to both individuals and sites. It was clear that careful consideration is needed before labeling a site or institution a Genomic Observatory. While some institutions with clearly identified sites and observatory programs might already ‘brand themselves’ as GOs (e.g., the Moorea Ecostation), it might take longer for others to reach the needed institutional approvals. Modeled on other networks, it was suggested that the GOs Network should draft a “Directors Letter” outlining the goals and mission of the Network. GOs champions could give this letter to the directors of their institutions, perhaps leading to a Memorandum of Understanding, where an institution would effectively join the GOs Network and identify their study site/s as GOs. Along these lines, a draft Mission Statement was discussed and it was agreed that this was a good start and should be the basis for further consultation and refinement along with a draft Founding Charter:

#### Initial draft of a GOs Network Mission Statement

- To build a global network of premier research sites working to generate genomic biodiversity observations that are well-contextualized and compliant with global data standards.
- To encourage long-term, place-based, DNA-centric programs that quantify biotic interactions in an ecosystem and develop models of biodiversity to predict the quality and distribution of ecosystem services.
- To provide training, technical assistance, resources, and best practice guides as a learning platform for scientists and organizations wishing to carry out place-based genomic observations, particularly those in developing countries with high and/or vulnerable biodiversity.

**Findings:** The session identified a core set of “GOs Champions” to take forward the deliberations and continue launching the GOs Network. A group of representatives from candidate GOs sites volunteered and this core group was tasked with formulating the strategy for the Network and preparing for GSC 15 (Maryland, USA), GSC 16 (Brisbane, Australia) and other future GOs meetings, which would serve to broaden the geographic representation of GOs. This session provided critical face-to-face discussion and the group spent a significant amount of the time exploring shared interests and priorities. It was further agreed that in rolling out this phased launch the GOs Network would aim to build momentum and actively recruit further sites. The currently involved sites are “pillar sites” that already have significant genomic studies underway, but the GOs Network must evolve to be more balanced geographically, geopolitically, and scientifically. For example it should include more sites outside the US and Europe, more terrestrial sites, etc. The initial focus on “model systems” (pillars) yields many benefits including acceleration of scientific returns when a particular site is studied in detail, including the genomic layer of biodiversity. The session participants considered it important that the GOs Network should be open to sites that are just being established, as well as targeting sites that already have rich scientific legacies but which are just starting genomic research. The group also agreed that in terms of priorities, the GOs Network should aim initially at sharing experiences, techniques, know-how, best practices, and standards to better study each individual site. As the community matures, however, it will become a distributed research platform (instrument) able to utilize these shared approaches to address joint scientific questions that require coordinated study across a diversity of sites.

Finally, it was recognized that Biodiversity Genomics is one of the unifying themes of the GOs Network and that this should be clearly articulated in future refinements of the Mission Statement and Founding Charter for discussion/validation with the broader community.

**Actions:** A final wrap-up session was chaired by Linda Amaral-Zettler (Marine Biological Laboratory Woods Hole, USA). Priority actions immediately following GSC 14 were to write the GSC 14 meeting report, plan GSC 15 and the second GOs meeting (GOs2), review the Draft Founding Charter, formalize the concept of membership, take ownership of GSC project descriptions and support the working groups in defining further activities and priorities.

**Deliverables**: The key deliverables to be generated by the core group include a Founding Charter and Mission statement and a mechanism for formalizing the Network through definitions of membership.

## Formal Close of GSC 14

At the close, the GSC 14 workshop organizers handed over to the GSC 15 workshop organizers. The main meeting was adjourned and after lunch the MG4U stakeholder workshop and the GSC hackathon both started.

### Satellite 1. GOs Stakeholders workshop.

The workshop had the title “Potential of Genomics Technology for Marine Monitoring and the Marine Strategy Framework Directive (MSFD)”, and was arranged in collaboration with MG4U [6]. The meeting was organized by Matthias Obst (University of Gothenburg, Sweden) and chaired by John Benzie (University College Cork, Ireland), and assembled 31 genomics scientists as well as representatives of marine policy bodies. The program featured short scientific talks, breakout sessions and panel discussions [35].

**Introduction:** The Marine Strategy Framework Directive (MSFD) is a legal framework that demands a repertoire of knowledge from the European member states centered around the descriptors of ‘good environmental status’ (GES). The descriptors, together with associated ‘criteria’ and ‘indicators’ will be used to decide on the status of marine ecosystems, and how GES can be achieved and maintained in the future. On this basis, the member states have to provide the ‘initial assessment’ (of current status) and national determination of GES for their waters, including the nationally relevant targets and indicators. The member states are currently in the process of reporting these marine indicators to the Commission, and these will be the basis for their national marine monitoring programs from 2014 onwards. Currently there is a strong focus on three issues, (i) data, (ii) methods to turn data into knowledge, and (iii) boundaries between ‘good’ and ‘no good’ status. There are still significant knowledge gaps in the understanding of marine ecosystems, especially when following an ecosystem-based approach. Furthermore, in many cases important baseline knowledge that is necessary to define the GES of European marine waters is missing. Currently there are no genomic (many genes across genome) and few genetic (single or small number of genes or markers) methods considered for contribution to the MSFD indicators.

**Goals:** The goal of the workshop was to discuss how genetic information and analytical methods can contribute to cost-efficient monitoring of marine ecosystems. The workshop was used in particular to identify genomic methods that can contribute knowledge to the descriptors of the MSFD, and to discuss how genomic methods can be integrated into marine monitoring programs.

**Findings:** Genomic methods have a high potential to provide ecosystem-based knowledge in multiple areas of marine monitoring, such as providing a consistent and high quality standard of species level identification, generating biodiversity estimates that include all branches of life, providing estimates of ecosystem function at all biological levels, discovering ecosystem wide trophic interactions, and estimating genetic diversity at the population level. The group concluded that genomic methods with high potential fall into four categories:

- Methods that generate the same knowledge faster, cheaper, and/or better compared to conventional methods (*e.g*., species identification using marker genes such as DNA barcoding); qPCR for water quality assays; microarrays for detection of Harmful Algal Blooms - HABs)
- Methods that allow us to do things we could not do before (*i.e*., give us new types of data and knowledge, such as metagenomics to study the biodiversity and function of whole ecosystems)
- Methods that allow us to go from patterns to processes and unravel causalities (*e.g.,* transcriptional response of species to chemical exposure)
- Methods that have no alternative aside from molecular methods (e.g., SNPs for tracing populations of species and DNA barcoding for analyzing food webs)

It was agreed that genomic methods have a high potential to address many descriptors in a standardized way. Also, the fact that there are currently no genetic or genomic approaches in the monitoring programs will make it relatively straightforward to introduce the standards developed by the GSC into Marine Monitoring.

The methods that meet the above criteria, are routinely established, and have existing pilots are (i) qPCR, (ii) DNA barcoding using marker genes, (iii) SNPs, and (iv) microarrays. These methods are cost-efficient and able to add significant knowledge to descriptors, including: D1 (Biological diversity), D2 (Non-indigenous species), D3 (Populations of fish and shellfish), D4 (Food webs), and D5 (Eutrophication), and D6 (Seafloor integrity). There are more methods with high potential in development, such as microbial metagenomics and transcription analysis, among others.

**Recommendations:** The entry point for these methods into regular monitoring programs should be at the national level, and so genomic scientists should partner with national institutes that currently implement the MSFD indicators. At the same time, a strong network should be developed in order to communicate the benefit of genomic tools to national environmental agencies, and to design pilot programs on the national and regional level. The network should include programs like the COST action EMBOS, the Micro B3 action Ocean Sampling Day, the FP7 project DEVOTES, the Genomic Observatories Network, the Genomics Standard Consortium, the EMBRC infrastructure, FP7 Project STAGES, and European marine GEO-BON initiatives.

**Action:** As a follow up to the stakeholder workshop, MG4U will circulate and assemble a white paper entitled ‘Genomics in marine monitoring: new opportunities for assessing marine health status’, including guidelines for the integration process of genomic methods into ongoing monitoring programs. This roadmap will contain a summary of methods that have the potential to contribute to or generate new knowledge mapped against MSFD indicators, and a cost-benefit comparison (based on existing case studies that use these methods), as well as a discussion of analytical approaches. In addition, the white paper will provide an overview of infrastructures needed for genomic monitoring (e.g., GOs Network) and stakeholders involved, such as national environmental agencies working with the MSFD, and also Regional Seas Conventions and the EU commission. The paper will be used as a guiding document for communication with organizations involved in monitoring.

**Deliverables:** MG4U and GSC will deliver the white paper ‘Genomic Methods in Marine Monitoring and the Marine Strategy Framework Directive (MSFD)’.

As a follow up to the stakeholder workshop, MG4U will circulate and assemble a white paper entitled ‘Genomic Methods in Marine Monitoring and the Marine Strategy Framework Directive (MSFD)’, with guidelines for the integration process of genomic methods into ongoing monitoring programs. Genomic methods have a high potential to provide ecosystem-based knowledge in multiple areas of marine monitoring, such as providing a consistent and high quality standard of species level identification, generating biodiversity estimates that include all branches of life, providing estimates of ecosystem function at all biological levels, discovering ecosystem wide trophic interactions and estimating genetic diversity at the population level. The methods identified, such as barcoding, amplicon sequencing, microarrays, quantitative real-time polymerase chain reaction (qRT-PCR), short nucleotide polymorphisms (SNP’s), transcriptomics and metagenomics can contribute to developing ‘next generation’, integrated, monitoring approaches.

The roadmap will include a summary of methods that have the potential to contribute to or generate new knowledge mapped against MSFD indicators, and a cost-benefit comparison (based on existing case studies that use these methods), as well as a discussion of analytical approaches. In addition, the white paper will provide an overview of infrastructures needed for genomic monitoring (*e.g.,* GOs Network) and stakeholders involved, such as national environmental agencies working with the MSFD, and also Regional Seas Conventions and the EU commission.

This paper will be used as a ‘living document’ for communication with organizations involved in monitoring.

**Deliverables:** MG4U and GSC will deliver the guideline paper ‘Genomic Methods in Marine Monitoring and the Marine Strategy Framework Directive (MSFD)’ with a roadmap for the integration of genomic methods in marine monitoring programs.

## GSC Biocode Commons Hackathon

The GSC has previously hosted two small hackathons offering a chance for technical work to take place on specific topics. This was the first GSC meeting to include a hackathon. Two topics were taken forward. John Deck (University of California Berkeley), USA led a group on “Building a Reference Model for Bio-Collections” and Renzo Kottman (Max Planck Institute Bremen, Germany) led a group on Field Information Management Systems (FIMS) using OSD and the Euromarine project as Use Cases.

Participants in the first hackathon included John Deck (University of California Berkely, USA), Norman Morrison (University of Manchester, UK), Sujeevan Ratnasingham (University of Guelph, Canada), Phillipe Rocca-Serra (University of Oxford, UK), Barry Smith (University of Buffalo, USA), Ramona Walls (New York Botanical Garden, USA), Trish Whetzel (Stanford University, USA), John Wieczorek (University of California Berkeley, USA), Jai Rideout (Northern Arizona University, USA), Matthew Bietz (University of Washington, USA), Steven Stones-Havas (Biomatters Ltd., New Zealand), Gabriele Dröge (Free University of Berlin, Germany), Éamonn O’Tuama (Global Biodiversity Information Facility, Denmark), Peter Sterk (University of Oxford, UK), Robert Robbins (University of California San Diego), Pier Buttigieg (AWI, Jacobs University Bremen, Germany), Reed Beaman (University of Florida, USA), and Michelle Koo (University of California Berkeley, USA). Participants in the second hackathon: Juliane Casquet (CNRS & University of Toulouse, France), Julia Schnetzer (Max Planck Institute Bremen, Germany), Steve Stones-Havas, John Deck, Jai Rideout, Matthew Bietz, Robert Robbins, Norman Morrison (University of Manchester, UK). Éamonn O’Tuama, Peter Sterk, Philippe Rocca-Serra, Pier Luigi Buttigieg, Evangelos Pafilis (HCMR Crete, Greece), Giorgos Kotoulas (HCMR Crete, Greece), Linda Amaral-Zettler (Marine Biological Laboratory, Woods Hole, USA), Neil Davies (University of California Berkeley, French Polynesia), Gabi Dröge, Chris Meyer (Smithsonian Institution, USA), Mesude Bicak (NERC Centre for Ecology and Hydrology Wallingford, UK), Inigo San Gil (University of New Mexico / LTER, USA), Pelin Yilmaz (Max Planck Institute Bremen, Germany), Anna Klindworth (Max Planck Institute Bremen, Germany), and Ramona Walls.

### Building a Reference Model for Bio-Collections Hackathon

The first hackathon built a reference model for bioCollections, using Protégé [36] to model this within the basic formal ontology framework [37] with the resulting product posted to the Biocode Commons Google Code site [38]. Primary contributions to this ontology included definitions for types of sampling processes, including, for instance, identification process, observing process, sub-sampling process (a complete list of processes can be found by examining the ontology file posted on the Google Code site. In addition to the ontology output we proposed expanding the Darwin Core “Basis of Record” to include DNAExtract and Subsampled Tissue in order to align Darwin Core with what we discussed. Several further actions were identified that could be addressed at future events.

### Field Information Management Systems Hackathon:

The second hackathon dealt with "Methods linking data at the point of sampling & collection" as part of EuroMarine [39]. The focus was on advancing technological developments to consistently track samples and contextual data to link multidisciplinary data from the point of collection. The two goals were to (i) track samples using online databases synchronized with offline field/lab applications (spreadsheets) that are compatible with data collection across the fields encompassed in EuroMarine and (ii) enable linking multidisciplinary data from the point of collection using for example geo-references and supporting structured storage and electronic exchange, thus reducing the need for retrospective text and data mining. This hackathon was a follow up of the EuroMarine Task 4.2 expert workshop held on 29^th^ September 2011 at the Max Planck Institute for Marine Microbiology in Bremen, Germany. All participants using or developing field information management systems (FIMS) introduced their tools and approaches and detailed on the efforts to develop and maintain the tools. This laid the foundation to create a concept for a mobile device (a smartphone application) able to support the data acquisition and distribution. It was decided to create such an app first for Android phones and later for IPhones and use the upcoming pilot Ocean Sampling Days [29] of the Micro B3 Project [7] as use cases. The complete protocol of the Hackathon is available at http://www.euromarineconsortium.eu/downloads/category/17-sampling%20and%20data%20collection?download=156:euromarine-t4-2-report-05-11-2012-final.

## Conclusions

In conclusion, this was one of the largest GSC meetings to date and served to launch the GOs Network (GOs1 meeting). There was strong support from a range of candidate genomic observatories (Table 1) and GOs Partners (Table 2). There was discussion of potential GOs Actions and Ocean Sampling Day is building momentum. This meeting identified a GOs core group of champions, with an overarching focus on biodiversity genomics and at least one working group in four key areas: science, informatics, technologies, and policy. The GOs core group will work towards a mission statement, a founding charter, defining membership, and recruitment of further GOs and Partners. The GOs Network will continue to incubate in the GSC for the foreseeable future.
